# Optimization of process parameters for trimethoprim and sulfamethoxazole removal by magnetite-chitosan nanoparticles using Box–Behnken design

**DOI:** 10.1038/s41598-023-41823-w

**Published:** 2023-09-02

**Authors:** Mahsa Alishiri, Maryam Gonbadi, Mehdi Narimani, Seyyed Amirreza Abdollahi, Negin Shahsavaripour

**Affiliations:** 1 Department of Biomedical Engineering, University of Illinois Chicago, Chicago, USA; 2 Nanochemical Engineering Department, Faculty of Advanced Technologies, Shiraz University, Shiraz, Iran; 3 Faculty of Mechanical Engineering, University of Tabriz, Tabriz, Iran; 4 Technical and Engineering Faculty, Islamic Azad University, Sirjan, Iran

**Keywords:** Environmental sciences, Chemistry

## Abstract

The contamination of the aquatic environment with antibiotics is among the major and developing problems worldwide. The present study investigates the potential of adsorbent magnetite-chitosan nanoparticles (Fe_3_O_4_/CS NPs) for removing trimethoprim (TMP) and sulfamethoxazole (SMX). For this purpose, Fe_3_O_4_/CS NPs were synthesized by the co-precipitation method, and the adsorbent characteristics were investigated using XRD, SEM, TEM, pH_zpc_, FTIR, and VSM. The effect of independent variables (pH, sonication time, adsorbent amount, and analyte concentration) on removal performance was modeled and evaluated by Box–Behnken design (BBD). The SEM image of the Fe_3_O_4_/CS adsorbent showed that the adsorbent had a rough and irregular surface. The size of Fe_3_O_4_/CS crystals was about 70 nm. XRD analysis confirmed the purity and absence of impurities in the adsorbent. TEM image analysis showed that the adsorbent had a porous structure, and the particle size was in the range of nanometers. In VSM, the saturation magnetization of Fe_3_O_4_/CS adsorbent was 25 emu g^−1^ and the magnet could easily separate the adsorbent from the solution. The results revealed that the optimum condition was achieved at a concentration of 22 mg L^−1^, a sonication time of 15 min, an adsorbent amount of 0.13 g/100 mL, and a pH of 6. Among different solvents (i.e., ethanol, acetone, nitric acid, and acetonitrile), significant desorption of TMP and SMX was achieved using ethanol. Also, results confirmed that Fe_3_O_4_/CS NPs can be used for up to six adsorption/desorption cycles. In addition, applying the Fe_3_O_4_/CS NPs on real water samples revealed that Fe_3_O_4_/CS NPs could remove TMP and SMX in the 91.23–95.95% range with RSD (n = 3) < 4. Overall, the Fe_3_O_4_/CS NPs exhibit great potential for removing TMP and SMX antibiotics from real water samples.

## Introduction

Antibiotics are a large group of pharmaceutical substances that kill bacteria or slow their growth. They have extensive applications in medical infection treatment, accounting for approximately 15% of total drug consumption^1–3^. An important point to consider is that less than 10% of these drugs undergo any transformation in the body, while the rest are excreted unchanged^4–6^. Antibiotics can find their way into the environment through various pathways, including discharge from manufacturing sites, domestic and hospital wastewater, gradual disposal of expired medications, topical application of drugs, and washing off drugs from the skin or contaminated clothes^7–9^.

The combination of trimethoprim (TMP) and sulfamethoxazole (SMX) as cotrimoxazole is present in several pharmaceutical forms, including oral suspension, tablets, and intravenous infusion for human or veterinary use. This combination is widely used to treat urinary tract and respiratory infections^10–12^. Environmental issues due to releasing TMP and SMX antibiotics into water sources pose a serious threat. Their most significant impact lies in the toxicity to micro-organisms present in the environment, disrupting ecological balance^13^. TMP and SMX antibiotics can also be used as liquid manure or sewage sludge fertilizers, entering aquatic environments, soil, and the food chain, leading to microbial resistance against them^14^. Consequently, drug resistance may occur in individuals, making common antibiotics ineffective in responding to infections in the body^15^.

Several methods have been employed for the removal of antibiotic from water. These techniques include reverse osmosis, ion exchange, coagulation, membrane filtration, and adsorption^16–19^. However, specific approaches have not been considered due to their associated limitations, including the need for extensive maintenance, high energy, chemical consumption, and suboptimal efficiency. Adsorption is one of the most widely used techniques in wastewater treatment processes due to its simplicity and relatively low cost^20^. During adsorption, dissolved ions or compounds are adsorbed and remain on the adsorbent solid surface^21^.

Recently, nanomaterials have garnered significant attention as exceptional adsorbents, primarily due to their high specific surface area and numerous active groups. Additionally, certain nanoparticles can selectively adsorb specific compounds, making them highly versatile for diverse chemical groups^22,23^. Over the past two decades, research on the exceptional electrical, catalytic, optical, and magnetic properties of nanoparticles has progressed rapidly^24^. Among the different types of nanoparticles, metal nanoparticles have attracted considerable attention due to their unique physical and chemical properties, including their ability to penetrate into microscopic pores, colloidal stability, and resistance to aggregation in solutions^25,26^.

As a subset of metal nanoparticles, magnetic nanoparticles can be easily separated from solutions using an magnet, eliminating the need for centrifugation and filtration. Hematite (α-Fe_2_O_3_), magnetite (Fe_3_O_4_), and maghemite (γ-Fe_2_O_3_) are important members of the Iron family because of their anti-corrosion properties, adjustable optical and magnetic characteristics, excellent chemical stability, cost-effectiveness, and environmental friendliness^27,28^. Among the adsorbents, magnetite-chitosan nanoparticles (Fe_3_O_4_/CS NPs) have been used to remove pollutants from aqueous environments because of the high potential, high adsorption capacity, and high specific surface area of these nanoparticles^29,30^.

In a study, Karimi et al. used Fe_3_O_4_/CS nano-bio-adsorbent to remove Cd (II), Cu (II), and Zn (II) ions from an aqueous solution. The removal rate for Cd (II), Cu (II), and Zn (II) ions was 99.98%, 93.69%, and 83.81%, respectively, at pH of 5.3, a metal concentration of 10 mg L^−1^, the adsorbent dosage of 1 g L^−1^, and room temperature for 24 h^31^. Thinh et al. used Fe_3_O_4_/CS NPs to remove Cr (VI) from the aqueous solution. The results revealed that the highest adsorption capacity of 55.80 mg g^−1^ was obtained at a pH of 3 for 100 min at room temperature^32^. In another study, Shen et al. evaluated the removal of C. I. Acid Red 73 by magnetic chitosan-Fe (III) hydrogel. This study obtained the maximum adsorption capacity of 294.5 mg g^−1^ in 10 mL at a dosage of 0.02 g, a pH of 12, and 25 °C with a concentration of 50 mg L^−1^^33^.

Nowadays, design of experiments (DOEs) such as response surface methodology (RSM) are used instead of classical methods (one-factor-at-a-time)^34^. RSM is a combination of mathematical and statistical methods used in process optimization, where many variables affect the desired response. This method can be performed to lower the number of tests, determine square regression coefficients, and evaluate the relationship between one or more responses using the influence of independent variables^35,36^.

Therefore, the present study aims to investigate the efficiency of Fe_3_O_4_/CS adsorbent to remove TMP and SMX from aqueous solutions. Further, the effect of pH, sonication time, adsorbent amount, and analyte concentration on the removal efficiency of contaminants is investigated. Analysis of variance (ANOVA) was used as a statistical method to analyze the responses. Moreover, BBD-based RSM was employed to achieve appropriate response levels for optimizing the removal of contaminants from aqueous solutions. Overall, this design has several advantages, such as reducing the number of tests, time, and cost and increasing removal efficiency.

## Materials and method

### Reagents and materials

All chemicals used in the experiments were of analytical grade. Chitosan was purchased from Aladdin Chemical Reagent Co. Ltd. Also, Iron (II) chloride, tetrahydrate, iron (III) chloride hexahydrate, trimethoprim, sulfamethoxazole, ethanol, acetone, hydrochloric acid, ammonia, acetic acid, sodium hydroxide, acetonitrile, and nitric acid were obtained from Sigma Aldrich Co. Ltd. Distilled water was used in all experiments to increase the volume of the solutions. The stock solution was prepared from each antibiotic with a concentration of 1000 mg L^−1^, and pH was determined via a pH-Meter. The ultrasonic bath was used to create interaction between the adsorbent and analyte. The samples containing antibiotics were analyzed by UV–Vis spectrophotometer. A centrifuge and a magnet were also used to separate the adsorbent from the solution. Adsorbent characteristics such as morphology, adsorbent shape, and phase characteristics were investigated using scanning electron microscopy (SEM), transmission electron microscopy (TEM), X-ray diffraction (XRD), pH of zero point of charge (pH_zpc_), vibrating sample magnetometer (VSM), and Fourier-transform infrared (FTIR).

### Synthesis of Fe_3_O_4_ NPs

Fe_3_O_4_ NPs were synthesized by the co-precipitation method. To this end, a mixture of 4 mL FeCl_3_ (2 M) and 2 mL FeCl_2_ (2 M) was prepared in a flat-bottom beaker. This solution was severely stirred for 30 min at 30 °C, and chemical precipitation was formed by slowly adding 100 mL of ammonia solution (1 M) to the mixture. The steps for this process were carried out under intense stirring and N_2_ gas. Black Fe_3_O_4_ NPs were separated by an external magnet. Finally, the NPs were rinsed with distilled water and ethanol until the pH reached 7.

### Synthesis of Fe_3_O_4_/CS NPs

Fe_3_O_4_/CS NPs were prepared using the co-precipitation method. To this end, 0.3 g of chitosan was dissolved in 50 mL of acetic acid solution (1%, V/V). Then, 2 g of Fe_3_O_4_ NPs prepared in the previous section were added to the mixture. The mixture was stirred for 30 min, followed by adding 50 mL of 1 M NaOH solution to the suspension to obtain Fe_3_O_4_/CS NPs. Finally, the prepared NPs were rinsed with distilled water until the pH reached 7 and then dried. The properties and morphology of the Fe_3_O_4_/CS NPs were investigated using SEM, VSM, pH_zpc_, TEM, FTIR, and XRD analyses.

### pH of zero point of charge (pH_zpc_)

The pH_zpc_ is the point at which the surface charge of the adsorbent is neutral. It is one of the crucial stages in determining the surface characteristics of the adsorbent. For this purpose, 10 Erlenmeyer flasks (100 mL) containing 30 mL of NaCl solution (0.01 M) were prepared, and each Erlenmeyer flask was adjusted to different pH values ranging from 2 to 11. The pH adjustments were performed using NaOH (0.1 M) and HCl (0.1 M). Subsequently, 0.2 g of the adsorbent was added to each Erlenmeyer, and the Erlenmeyers were placed on a shaker for 24 h. After 24 h and the separation of the adsorbent from the solution, the final pH was measured. The difference between the initial and final pH values was calculated, and the ΔpH curve was plotted against the initial pH values. The point where the curve intersects the X-axis is called the pH_zpc_.

### Response surface methodology (RSM)

RSM applies experimental techniques to design and optimize the relationships between the test factors. Next, after checking the responses, it analyzes and presents graphs based on one or more criteria^37^. In this research, pH solution (A), analyte concentration (B), adsorbent amount (C), and sonication time (D) were selected as parameters affecting the removal performance of antibiotics. Design-Expert^®^ software (version 10) was used to examine the effect of parameters on response performance (removal efficiency). Then, experiments were designed using Box–Behnken Design. The number of experiments was determined using Eq. (1).

1 $$ {\text{N}} = {\text{2K }}\left( {{\text{K}} - {1}} \right) \, + {\text{ C}}_{{\text{o}}} $$

where *K* is the number of investigated parameters and *C*_*o*_ is the number of repetitions of the experiment steps^38^.

A total of 29 experiments (with 5 central replicates) were designed and implemented for each analyte. After selecting the model, the equation of the model and its predicted coefficients were determined through the quadratic equation (Eq. 2):

2 $$\mathrm{Y }={\beta }_{0}+\sum_{i=1}^{k}{\beta }_{i}{X}_{i}+ \sum_{i=1}^{k}{\beta }_{ii}{X}_{i}^{2}+ \sum_{i\le j}^{k}\sum_{j}^{k}\beta ij{X}_{i}{X}_{j}$$

where, *β*_*0*_, *β*_*i*_, *β*_*ii*_, and *β*_*ij*_ are constant, linear, quadratic, and interaction coefficients of regression, respectively. Also, *X*_*i*_ and *X*_*j*_ denote coded independent variables, and *k* is the number of variables^39^. Statistical analysis was run to evaluate the accuracy and adequacy of the model using ANOVA, with probability values of Prob < F > 0.05. Further, the predictability and adequacy of the model were tested using the coefficient of determination of linear regression (R^2^), adequate precision, the lack of fit criterion, adjusted determination coefficient (Adj-R^2^), and detect the residuals. For the experimental design in the current study, four factors were selected, including pH, adsorbent amount, antibiotic concentration, and sonication time. Each factor was considered at three levels, as shown in Table 1.

**Table 1:** The BBD based on RSM.

Variables	Symbol	Unit	Range and levels		
			− 1	0	+ 1
pH of the solution	A	–	3	6	9
Analyte concentration	B	mg L^−1^	10	20	30
Adsorbent amount	C	g	0.05	0.10	0.15
Sonication time	D	min	10	20	30

### Adsorption experiments

All adsorption experiments were done in a discontinuous system. For this purpose, 100 mL of solutions containing different concentrations of antibiotics were prepared in 250 mL Erlenmeyer flasks. Moreover, pH in the range of 3 to 9 was investigated to examine the effect of pH on the removal of analytes. Then, 0.05–0.15 g of Fe_3_O_4_/CS NPs was added to the solutions containing the analytes. The solutions prepared based on the model provided by RSM were placed in the ultrasonic bath, and the time was determined for each test by the software. Afterward, the adsorbent was separated from the sample using centrifugation and an external magnetic field. In the next step, the concentration of antibiotics was measured by UV–Vis spectrophotometer. Finally, the removal efficiency was calculated using Eq. (3).

3 $$\mathrm{\%R }=\frac{{\mathrm{c}}_{\mathrm{o}}-{\mathrm{c}}_{\mathrm{e}}}{{\mathrm{c}}_{\mathrm{o}}}\times 100$$

In this equation, *C*_*o*_ (mg L^−1^) was the initial concentration of the analyte in the solution, *C*_*e*_ (mg L^−1^) was the equilibrium concentration of the analyte after the equilibration time, and *R* was the removal efficiency^40^.

## Results and discussion

### Characterization of Fe_3_O_4_/CS NPs

**Figure 1:**
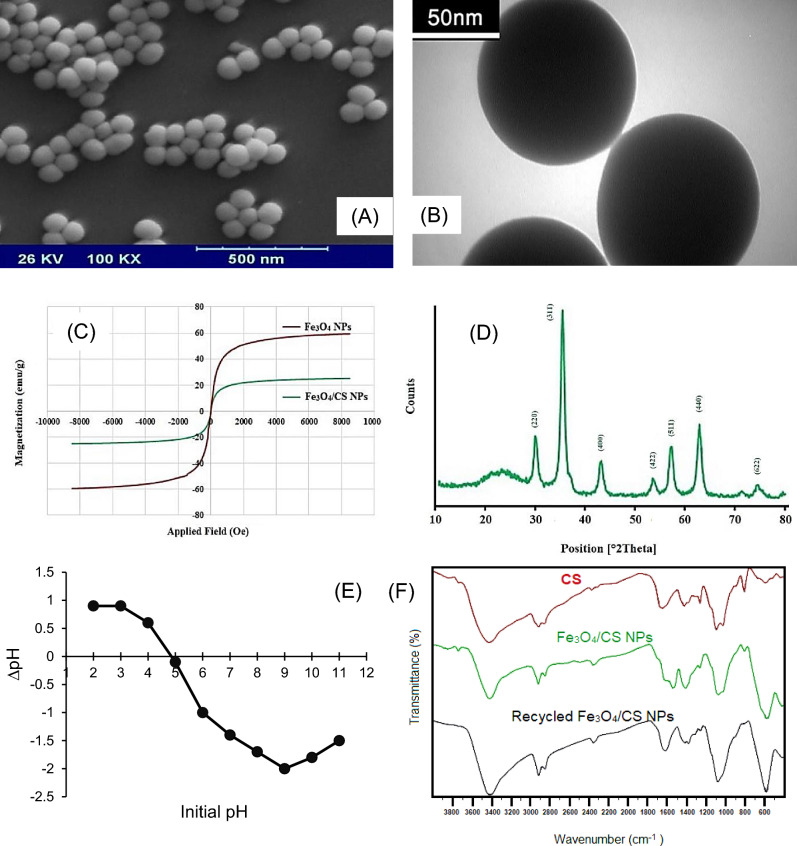
(**A**) SEM image, (**B**) TEM image, (**C**) VSM magnetization curve, (**D**) XRD pattern, (**E**) pH_zpc_, and (**F**) FTIR analysis of Fe_3_O_4_/CS NPs adsorbent and after 6 cycles of sorption desorption processes.

SEM images were captured for the Fe_3_O_4_/CS sample to examine the size of nanoparticles and their morphology (Fig. 1A). Figure 1A shows a spherical morphology and uniformity for the sample, along with appropriate granulation with no aggregation. In addition, particle size was determined between 75 and 81 nm. Figure 1B illustrates the TEM image of Fe_3_O_4_/CS adsorbent. The TEM image in Fig. 1B confirms the sample’s spherical morphology and uniform size. Moreover, the magnetic property of the synthesized Fe_3_O_4_/CS sample was tested by the VSM method at room temperature (Fig. 1C). The magnetic diagram for the sample is similar to the letter S, and the curved shape of the sample confirms the superparamagnetic property of the sample. In addition, the degree of saturation of the sample was equal to 25 emu g^−1^. This value suggests the high magnetic property of the sample and its easy separation from the reaction environment by an external magnet. The magnetic property of the sample was compared by performing VSM analysis for the pure iron oxide nanoparticles. For the pure Fe_3_O_4_ sample, the saturation magnetization was 59 emu g^−1^, which is much higher than that of the Fe_3_O_4_/CS composite sample. This result indicates the presence of chitosan next to iron oxide nanoparticles and the change of iron percentage in the sample compared to its pure ratio. XRD analysis was performed to identify and confirm the structure of Fe_3_O_4_/CS crystals. For the Fe_3_O_4_/CS sample, the position and relative intensity of all peaks according to the standard XRD pattern for Fe_3_O_4_ NPs is JCPDS card no. 1436-85^41^. The presence of sharp peaks with high intensity indicates a good network structure. On the other hand, the absence of additional peaks confirms the purity and the absence of impurities in the produced sample (Fig. 1D). Seven peaks with angles of 30.2°, 35.8°, 43.5°, 53.7°, 57.4°, 62.8°, and 75.3° and corresponding crystal plates of 220, 311, 400, 422, 511, 440, and 622, respectively, were identified in the spectrum. These peaks are related to iron oxide nanoparticles with a cubic spinel lattice structure. Moreover, the weak broad peak that appeared at the angle of 21–28 is related to chitosan, which is present in the substrate of the magnetic composite sample. The size of Fe_3_O_4_/CS crystals was about 70 nm using the Debye–Scherrer Equation. The results of pH determination showed that the Fe_3_O_4_/CS adsorbent surface has zero electric charge at pH = 4.9 (Fig. 1E). Therefore, the adsorbent surface will have a positive electrical charge at a pH lower than pH_zpc_ and a negative electrical charge at a pH higher than pH_zpc_. FTIR spectroscopy was used to identify and confirm the structure of Fe_3_O_4_/CS NPs. For this purpose, FT-IR analysis was carried out from two combinations of Fe_3_O_4_/CS NPs, CS. Besides, FT-IR analysis of Fe_3_O_4_/CS NPs was also performed after 6 cycles of sorption and desorption. In the FT-IR spectrum related to chitosan, the broad peak at 3427 cm^−1^ is related to the stretching vibrations of amine and hydroxyl groups on the chitosan chain, and the peaks at 2922 cm^−1^ and 2851 cm^−1^ are attributed to the stretching vibrations of carbon-hydrogen bonds. The bending vibrations related to the nitrogen–hydrogen bond appear at 1649 cm^−1^ and the peak at 1262 cm^−1^ is related to the carbon–oxygen stretching of the first type of alcohol in the chitosan chain. The double peaks at 1093 cm^−1^ and 1027 cm^−1^ are attributed to the carbon–oxygen stretching vibrations of the chitosan structure. The FT-IR analysis of Fe_3_O_4_/CS NPs shows well all the peaks related to chitosan with a slight shift, in addition to the sharp and distinct peak at 576 cm^−1^ related to the stretching vibration of the iron-oxygen (Fe–O) bond, which is added to it. It confirms the presence of iron oxide nanoparticles in the composition. Also, as shown in Fig. 1F, these peaks were again observed with full intensities like the initial absorbance when performing the desorption processes.

### Statistical analysis

Design Expert software was used for regression analysis, drawing RSM graphs, and ANOVA. ANOVA results are presented in Tables 2 and 3. The tables show the parameters and statistical analysis of the presented second-order model for removing TMP and SMX. The determination coefficient (R^2^) and adjusted determination coefficient (Adj-R^2^) were used to estimate the goodness of the fit of the model. The high value of R^2^ (i.e., 0.9993 for TMP and 0.9973 for SMX) indicates that the model can explain more than 99.7% of the variation. Also, the value of Adj-R^2^ more than 0.99 shows a high degree of correlation between the experimental and predicted values. Therefore, the closer the R^2^ and Adj-R^2^ are to 1, the better the model describes response changes as a function of independent variables. The significance of the model for removing the contaminants is expressed by F-value, which was 1433.03 and 373.25 for TMP and SMX, respectively. Adeq-Precision shows the difference between the model’s predicted response and the average value of the prediction error. When this criterion is greater than 4, it indicates the acceptable discrimination power of the model. In this study, Adeq-Precision for removing TMP and SMX was 113.73 and 65.61, respectively. Another parameter used to evaluate the model is the lack of fit (LOF) test. This test is significant if the *P*-value of the model is greater than 0.05 (95% significance level). Tables 2 and 3 show that the *P*-value for removing TMP and SMX is greater than 0.05. Quadratic equations (Eqs. 4 and 5) demonstrate the mathematical relationship between the parameters for removing TMP and SMX using RSM.

4 $$ \begin{aligned} \% {\text{Removal-TMP}} &=  + {88}.{23 } + {8}.{42}*{\text{A }} - {1}.{46}*{\text{B }} + {3}.{12}*{\text{C }} \hfill \\ & \quad  - {4}.{21}*{\text{D }} - {6}.{87}*{\text{A}}*{\text{B}} - {24}.{84}*{\text{A}}*{\text{C}} - {1}.0{2}*{\text{A}}*{\text{D}} \hfill \\  & \quad + {16}.{46}*{\text{B}}*{\text{C}} - {12}.0{1}*{\text{B}}*{\text{D}} - {7}.{42}*{\text{C}}*{\text{D}} - {25}.{13}*{\text{A}}^{{2}} \hfill \\ & \quad - {25}.{53}*{\text{B}}^{{2}} - {1}.{83}*{\text{C}}^{{2}} - {17}.{25}*{\text{D}}^{{2}} \hfill \\ \end{aligned} $$

5 $$ \begin{aligned} \% {\text{Removal-SMX}} & = + {93}.{14 } + {3}.{42}*{\text{A }} - {2}.{48}*{\text{B }} + {6}.{69}*{\text{C }} \hfill \\  & \quad- {5}.{3}0*{\text{D }} - {2}.0{2}*{\text{A}}*{\text{B}} - {17}.{75}*{\text{A}}*{\text{C}} + {3}.{61}*{\text{A}}*{\text{D}} \hfill \\  & \quad+ {9}.{79}*{\text{B}}*{\text{C}} - {4}.0{5}*{\text{B}}*{\text{D}} - {1}.{85}*{\text{C}}*{\text{D}} - {18}.{81}*{\text{A}}^{{2}} \hfill \\  & \quad- {15}.{75}*{\text{B}}^{{2}} - {6}.{12}*{\text{C}}^{{2}} - {12}.{77}*{\text{D}}^{{2}} \hfill \\ \end{aligned} $$

**Table 2:** ANOVA for response surface quadratic model for removal of TMP.

Source	Sum of squares	DF	Mean square	F-value	p-value	
Model	13,732.60	14	980.90	1433.03	< 0.0001	Significant
A	851.77	1	851.77	1244.37	< 0.0001	
B	25.87	1	25.87	37.80	< 0.0001	
C	117.31	1	117.31	171.39	< 0.0001	
D	212.69	1	212.69	310.72	< 0.0001	
A*B	188.93	1	188.93	276.01	< 0.0001	
A*C	2469.59	1	2469.59	3607.90	< 0.0001	
A*D	4.16	1	4.16	6.08	0.0272	
B*C	1083.73	1	1083.73	1583.25	< 0.0001	
B*D	577.20	1	577.20	843.25	< 0.0001	
C*D	220.67	1	220.67	322.38	< 0.0001	
A^2^	4098.83	1	4098.83	5988.09	< 0.0001	
B^2^	4228.65	1	4228.65	6177.76	< 0.0001	
C^2^	21.85	1	21.85	31.91	< 0.0001	
D^2^	1931.29	1	1931.29	2821.48	< 0.0001	
Residual	9.58	14	0.68			
Lack of fit	4.49	10	0.45	0.35	0.9175	Not significant
Pure error	5.09	4	1.27			
Cor total	13,742.19	28				
R^2^ = 0.9993	R^2^ adjust = 0.9986		Adeq-precision = 113.73			

*DF* degree of freedom.

**Table 3:** ANOVA for response surface quadratic model for removal of SMX.

Source	Sum of squares	DF		Mean square	F-value	p-value	
Model	6545.06	14		467.50	373.25	< 0.0001	Significant
A	140.43	1		140.43	112.11	< 0.0001	
B	74.25	1		74.25	59.28	< 0.0001	
C	538.01	1		538.01	429.54	< 0.0001	
D	338.03	1		338.03	269.88	< 0.0001	
A*B	16.40	1		16.40	13.10	0.0028	
A*C	1260.61	1		1260.61	1006.44	< 0.0001	
A*D	52.13	1		52.13	41.62	< 0.0001	
B*C	383.38	1		383.38	306.08	< 0.0001	
B*D	65.85	1		65.85	52.58	< 0.0001	
C*D	13.69	1		13.69	10.93	0.0052	
A^2^	2295.41	1		2295.41	1832.61	< 0.0001	
B^2^	1609.38	1		1609.38	1284.90	< 0.0001	
C^2^	243.67	1		243.67	194.54	< 0.0001	
D^2^	1059.28	1		1059.28	845.71	< 0.0001	
Residual	17.54	14		1.25			
Lack of fit	11.94	10		1.19	0.85	0.6200	Not significant
Pure error	5.59	4		1.40			
Cor total	6562.59	28					
R^2^ = 0.9973	R^2^ Adjust = 0.9947			Adeq-Precision = 65.61			

*DF* Degree of freedom.

The positive sign in front of the parameters displays the synergistic effect of the variable on the model. Meanwhile, the negative sign indicates a decreasing or opposite effect on the model. When an increase in the value of one variable is followed by an increase in the value of another variable, a positive correlation coefficient is obtained, which indicates synergy. On the other hand, when a reduction in the value of one variable is followed by a reduction in another variable, a negative correlation coefficient is obtained, indicating disintegration. Table 4 presents the experimental results and the results obtained from the Design-Expert^®^ software for removing TMP and SMX.

**Table 4:** BBD matrix for removal of TMP and SMX.

Run	Variables				%Removal-TMP		%Removal-SMX	
	A	B	C	D	Experimental	Predicted	Experimental	Predicted
1	0	− 1	− 1	0	75.98	75.67	76.11	76.84
2	0	0	1	1	60.21	60.63	73.78	73.77
3	− 1	1	0	0	34.84	34.54	53.88	54.70
4	0	1	1	0	78.55	78.98	86.51	85.26
5	1	0	− 1	0	91.58	91.40	82.37	82.68
6	0	0	− 1	1	68.70	69.23	63.29	64.08
7	0	1	− 1	0	40.10	39.81	53.54	52.29
8	− 1	0	− 1	0	24.13	24.86	41.07	40.33
9	0	0	0	0	89.48	88.23	92.37	93.14
10	− 1	0	0	− 1	40.15	40.60	66.49	67.05
11	1	− 1	0	0	54.09	54.33	67.19	66.51
12	1	0	1	0	48.75	47.96	59.46	60.57
13	0	0	0	0	87.36	88.23	93.71	93.14
14	0	0	− 1	− 1	63.28	62.80	70.85	71.00
15	1	0	0	− 1	58.99	59.49	67.28	66.67
16	1	1	0	0	37.09	37.65	56.55	57.49
17	− 1	0	0	1	34.61	34.22	49.12	49.21
18	0	0	0	0	88.01	88.23	94.53	93.14
19	0	0	1	− 1	84.50	83.91	88.74	88.09
20	0	0	0	0	86.99	88.23	93.57	93.14
21	0	1	0	− 1	60.31	60.20	71.03	71.49
22	0	− 1	0	1	54.66	54.72	65.94	65.85
23	0	− 1	0	− 1	38.88	39.11	68.26	68.35
24	0	− 1	1	0	48.59	49.00	69.92	70.66
25	0	1	0	1	28.04	27.75	52.48	52.76
26	1	0	0	1	49.37	49.03	64.35	63.28
27	0	0	0	0	89.32	88.23	91.54	93.14
28	− 1	0	1	0	80.69	80.81	89.17	89.23
29	− 1	− 1	0	0	24.35	23.73	56.42	55.62

**Figure 2:**
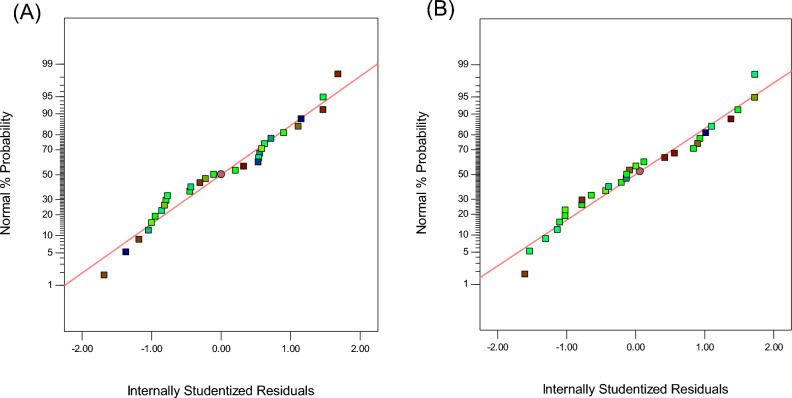
Normal plot of residuals for (**A**) TMP and (**B**) SMX.

**Figure 3:**
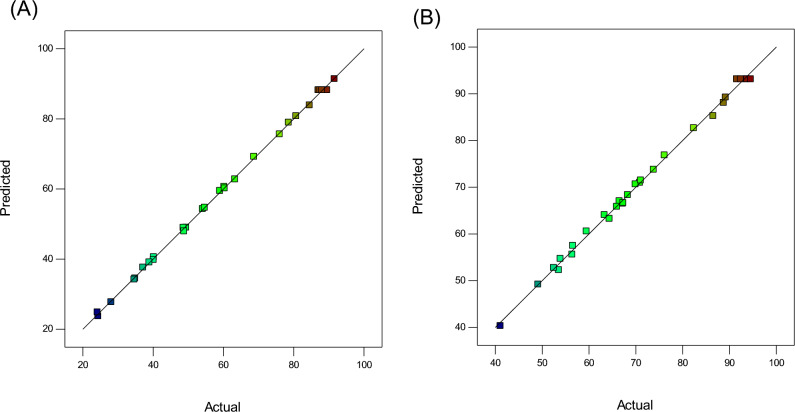
Graph of actual values versus predicted values for (**A**) TMP and (**B**) SMX.

**Figure 4:**
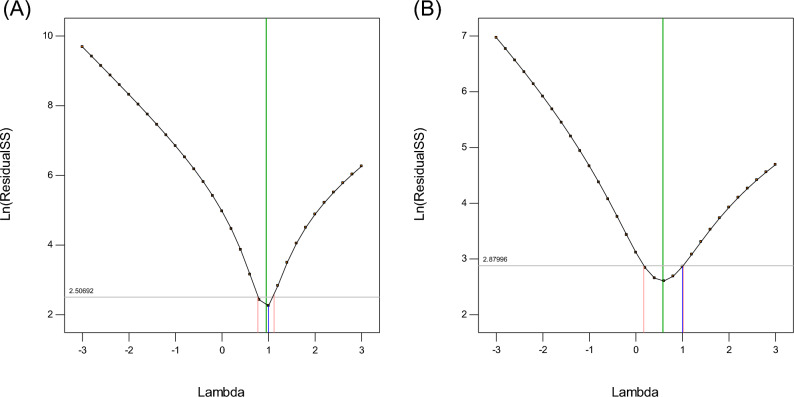
Box–Cox curve for (**A**) TMP and (**B**) SMX.

In addition to the mentioned criteria to evaluate the model’s accuracy, the difference between the experimental and predicted residuals was used to test the model’s accuracy (Figs. 2, 3, 4). The residuals are considered unfit changes by the model. Figure 2A and B present the residual diagram for evaluating the normal distribution of the residuals. The points in the normal graph of the residuals form a straight line, confirming the normal distribution of residuals.

Figure 3A and B display the actual versus predicted values for each model. The response values against the actual values are presented to help identify values or groups of values not predicted by the model. According to Fig. 3A and B, there is a trivial difference between predicted values and the experimental results.

Figure 4A and B provide Box–Cox curves. This figure is a tool to identify the most appropriate power transfer function to be applied to the response. The lowest point in the Box–Cox graph reflects the best Lambda value (Lambda: the minimum residual sum of squares in the transformed model). This figure also illustrates a 95% confidence interval. According to the Box–Cox graph, the value of Landa was considered equal to 1, and no transformation was needed. The graphs related to the adequacy of the model also exhibit the good performance of the model.

### Three-dimensional

**Figure 5:**
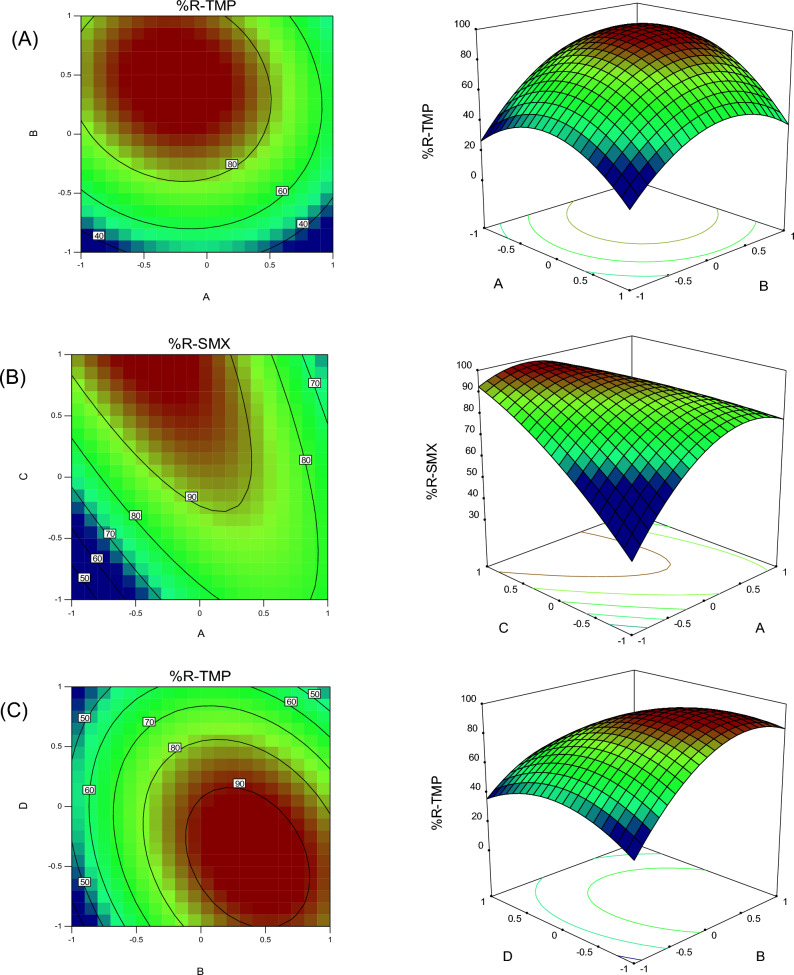
The three-dimensional (3D) plots of removal of (**A**) TMP, (**B**) SMX, and (**C**) TMP.

The effect of changes in TMP concentration on TMP removal efficiency in optimal conditions (pH of 6, adsorbent amount of 0.13 g/100 mL, and sonication time of 15 min) is presented in Fig. 5A. Changes in TMP concentration were investigated in the range of 10 to 30 mg L^−1^. The results (Fig. 5A) indicated that by keeping other factors (e.g., sonication time, adsorbent amount, and pH) constant in optimal conditions, the amount of TMP ion removal reduced with increasing concentration. As a result, the highest removal efficiency was observed at a concentration of 22 mg L^−1^. The explanation is that with the increase in the concentration of antibiotics in the solution, the competition for access to the binding sites from the adsorbent increases, and all the binding sites are exposed to analytes. Finally, it can be concluded that with the increase in concentration, the adsorbent surface is quickly saturated, and the removal percentage decrease. In other words, at low concentrations, more effective adsorption sites are available for antibiotics. In contrast, at higher concentrations, the number of antibiotics is much higher than that of adsorption sites on the adsorbent. Hence, the adsorption of cations depends on the initial concentration, and with increasing concentration, the absorption percentage decreases. Norzaee et al. investigated the removal of penicillin G antibiotics via persulfate. The results proved that the removal efficiency decreases with the increase in the initial concentration of antibiotics^42^. Similar results were obtained by Gao et al. on removing antibiotics from industrial wastewater by graphene oxide^43^.

Figure 5A illustrates the effect of pH on the removal efficiency of TMP by the adsorbent. The percentage of TMP removal increased as the pH changed from 3 to 6. Subsequently, at higher pH values, the removal of antibiotics decreased. The pH_zpc_ of Fe_3_O_4_/CS adsorbent was calculated to be 4.9. At pH values above the pH_zpc_, the surface of Fe_3_O_4_/CS adsorbent becomes negatively charged due to the presence of hydroxide ions (OH^-^), enabling the adsorption of antibiotics. The maximum removal for TMP and SMX was observed at pH = 6. It was assumed that at pH < 6, the high hydronium ions (H_3_O^+^) concentration competes with antibiotics, leading to reduced adsorption at lower pH levels. When the pH value was > 6, the increase in alkaline conditions and OH^-^ concentration in the solution resulted in electrostatic repulsion. Therefore, the removal efficiency decreased at higher pH values due to electrostatic repulsion. Wang and You investigated the removal of antibiotics from aqueous environments by iron oxide/activated carbon nanoparticle composite. The results showed that the removal rate of antibiotics is extremely low at a pH of less than 6. They explained this result by the high concentration of H^+^ ions in the experiment environment. These ions occupy the binding sites on the adsorbent surface and exhibit greater competition for surface adsorption^44^.

Figure 5B depicts the simultaneous effect of the two parameters (i.e., adsorbent amount and pH) on SMX removal. The results in Fig. 5B suggest that the analyte removal percentage increases with increasing the adsorbent amount. This outcome can be due to the rise in empty and unoccupied sites with increasing adsorbent content. In general, the surface adsorption of analytes increases with increasing the adsorbent since more adsorption sites will be available. When the amount of adsorbent is low, the removal percentage reduces, probably due to insufficient active sites and saturation of the adsorbent surface. In a study, Yang et al., using graphene oxide as an adsorbent to remove antibiotics from aqueous solutions, yielded results similar to those of the present research. Their results indicated that the removal efficiency increases with increasing the adsorbent amount^45^.

Figure 5C provides the effect of TMP concentration and reaction time on TMP removal efficiency. According to Fig. 5C, the removal efficiency increases by increasing the reaction time. The reason is that with the accumulation of analyte molecules in empty and unoccupied sites over time, the analyte concentration reduces, and the removal rate increases. Askari et al. investigated the removal of antibiotics from aqueous environments using walnut wood and magnetized with cobalt ferrite. The results revealed that the increase in time caused an increase in the removal percentage of antibiotics^46^. Similar results were obtained regarding the increase in removal percentage with increasing contact time in studies by Lanjwani et al., Vu et al., and Mohammed et al.^47–49^.

### Optimal points

The optimal points for removing the contaminants were obtained using the Design-Expert^®^ software, as reported in Table 5. The optimization is performed to find points with a higher removal index. This study optimized the important factors in removal efficiency (i.e., adsorbent amount, antibiotics concentration, sonication time, and pH solution). Table 5 represents the optimization results. It is of note that the experiments were done under optimal conditions to confirm the results obtained from the model prediction. The results indicated a good agreement with the removal value predicted by the model under optimal conditions.

**Table 5:** Optimum conditions of removal of TMP and SMX.

Run	A	B	C	D	%Removal-TMP		%Removal-SMX	
					Experimental	Predicted	Experimental	Predicted
1	6	22	0.13	15	93.85	93.51	95.98	97.14
2	6	22	0.13	15	93.40	93.51	97.24	97.14
3	6	22	0.13	15	92.46	93.51	98.01	97.14

### Desorption studies

**Figure 6:**
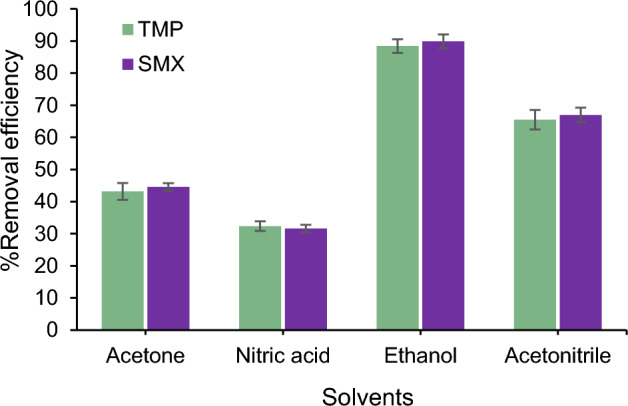
The effect of solvent on the desorption of analyte.

The desorption of the analyte from the adsorbent surface for its reuse is crucial. The desorption experiments of antibiotics from the Fe_3_O_4_/CS NPs surface were carried out using various solvents such as acetonitrile, acetone, ethanol, and nitric acid. These experiments were conducted under optimal conditions for the variables. After separating Fe_3_O_4_/CS NPs using an external magnetic field, the adsorbent surface was washed with the desired solvent. Finally, the remaining antibiotic concentration was measured using a UV/Vis spectrophotometer. Among four different solvents (ethanol, acetone, nitric acid, and acetonitrile), ethanol showed the highest desorption for TMP and SMX from the Fe_3_O_4_/CS NPs (Fig. 6). Therefore, ethanol was selected as the solvent in subsequent experiments.

### Reusability of Fe_3_O_4_/CS NPs

**Figure 7:**
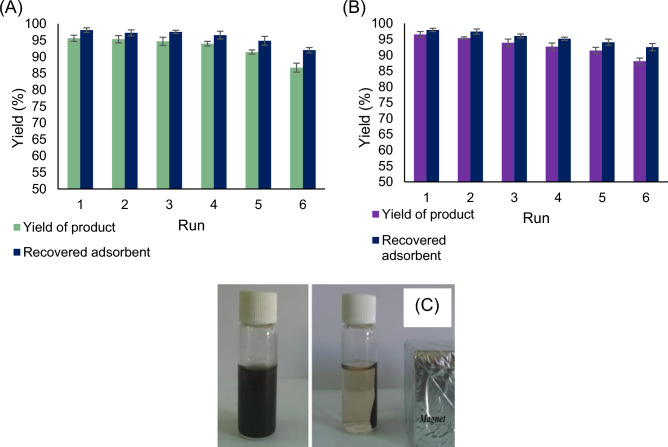
The reusability of adsorbent for removal of (**A**) TMP, (**B**) SMX, and (**C**) the removal illustration.

The expanded process of desorption has been adopted to reduce the production costs of the adsorbent and minimize the remaining waste materials. Based on the results, ethanol was selected as the most efficient solvent for the desorption of TMP and SMX from the adsorbent surface. Therefore, after the absorption of TMP and SMX from the aqueous solution with Fe_3_O_4_/CS NPs, the adsorbent was recovered and reused through six consecutive cycles of desorption with ethanol. Figure 7 illustrates the efficiency of desorption over the six stages of the recovery process. According to the results, the recovery percentage for TMP and SMX only decreased by about 5% after five cycles of absorption and desorption, indicating more than 95% recovery. The acceptable reduction in adsorption capacity signifies the remarkable stability of the adsorbent and the non-degradation of the Fe_3_O_4_/CS NPs structure, which highlights the economic feasibility of synthesizing and utilizing this adsorbent for the treatment of wastewater containing TMP and SMX. Figure 7C depicts the method of separating Fe_3_O_4_/CS NPs from the sample medium.

### Analysis of real samples

Several water samples (i.e., tap water, wastewater, and lake water fish farm) were used to test the validity and applicability of the proposed method. Then, TMP and SMX removal experiments were carried out on real samples under optimal conditions. To this end, water samples were filtered to remove suspended particles, and then experiments were performed according to the method stated in “Synthesis of Fe_3_O_4_/CS NPs” section. According to the results (Table 6), the removal efficiency of TMP and SMX in environmental water samples was in the range of 91.23%-98.28%, indicating that the sample texture has a trivial effect on the efficiency of the method.

**Table 6:** Results of removal of TMP and SMX in real samples (n = 3).

Real samples	%Removal-TMP ± RSD	%Removal-SMX ± RSD
Tap water	93.57 ± 2.07	95.14 ± 1.52
Fish farm	91.89 ± 3.09	91.23 ± 2.02
Wastewater	92.58 ± 2.30	95.20 ± 2.76
Lake water	95.95 ± 1.66	93.42 ± 1.80

### Comparison with other methods

The performance comparison of the proposed adsorbent with other adsorbents is shown in Table 7. The results of Table 7 showed that the proposed method was comparable to other methods available in the literature. On the other hand, the contact time for our research was superior to other adsorbents for removing TMP and SMX. The results showed that the ultrasonic-assisted removal method significantly improves the efficiency of the removal. Also, using the optimization method with the help of RSM reduces the number of tests and the consumption of materials.

**Table 7:** Literature comparison of removal for TMP and SMX.

Adsorbent	Analyte	Concentration (mg L^−1^)	Adsorbent amount	pH	Time	Adsorption capacity or %removal	Ref
Activated carbon F400	TMP	10	50 mg	7	30 min	70.6%	^50^
Granular activated carbon	SMX	25	4 g	7	100 min	94.23%	^51^
Amberlite XAD-7	TMP	50	3 g	7	120 min	70%	^52^
Hierarchical magnetic zinc oxide-based composite ZnO@g-C3N4	SMX	30	0.65 g	5.6	60 min	90.4%	^53^
Vegetal powdered activated carbon	TMP	15	100 mg	6	30 min	135 mg g^−1^	^54^
Fe-MIL-88B_NH_2_ carbonaceous nanocomposites	SMX	100	25 mg	6	12 h	73.53 mg g^−1^	^55^
Fe_3_O_4_/CS NPs	TMP	22	0.13 g	6	15 min	95.60%	This study
Fe_3_O_4_/CS NPs	SMX	22	0.13 g	6	15 min	95.16%	This study

## Conclusions

The present study used Fe_3_O_4_/CS NPs to determine the removal process of TMP and SMX. Accordingly, BBD-based RSM was applied to evaluate the effect of parameters on response performance. Independent variables, such as pH, sonication time, adsorbent amount, and analyte concentration, were optimized in removing TMP and SMX. The results of SEM, VSM, XRD, and TEM showed that the Fe_3_O_4_/CS NPs were successfully prepared and existed as spherical nanoparticles with an average size of about 70 nm. The large R^2^ coefficient guarantees the fit of the quadratic model and indicates good processing of the data under investigation. After optimization, the removal amount of TMP and SMX at a pH of 6, concentration of 22 mg L^−1^, adsorbent amount of 0.13 g/100 mL, and contact time of 15 min was 93.85% and 98.01%, respectively. The results of desorption studies showed that ethanol as a solvent increased the desorption efficiency of antibiotics from the adsorbent surface. Furthermore, reusability showed that Fe_3_O_4_/CS NPs could be used up to 6 times efficiently to remove TMP and SMX. The results also suggested that Fe_3_O_4_/CS NPs effectively removed TMP and SMX from real water samples and can remove TMP and SMX in the range of 91.23% to 95.95%. The results of real water samples analysis showed that the sample matrix had no significant effect on removing the TMP and SMX antibiotics from water samples. The experimental results demonstrated that Fe_3_O_4_/CS NPs could be employed successfully as environmentally friendly adsorbents for removing TMP and SMX antibiotics from the water samples.

## Data Availability

The datasets used and/or analyzed during the current study are available from the corresponding author upon reasonable request.
